# Fire Promotes Pollinator Visitation: Implications for Ameliorating Declines of Pollination Services

**DOI:** 10.1371/journal.pone.0079853

**Published:** 2013-11-12

**Authors:** Michael E. Van Nuland, Elliot N. Haag, Jessica A. M. Bryant, Quentin D. Read, Robert N. Klein, Morgan J. Douglas, Courtney E. Gorman, Trey D. Greenwell, Mark W. Busby, Jonathan Collins, Joseph T. LeRoy, George Schuchmann, Jennifer A. Schweitzer, Joseph K. Bailey

**Affiliations:** 1 Department of Ecology and Evolutionary Biology, University of Tennessee, Knoxville, Tennessee, United States of America; 2 Great Smoky Mountains National Park, Gatlinburg, Tennessee, United States of America; University of New South Wales, Australia

## Abstract

Pollinators serve critical roles for the functioning of terrestrial ecosystems, and have an estimated annual value of over $150 billion for global agriculture. Mounting evidence from agricultural systems reveals that pollinators are declining in many regions of the world, and with a lack of information on whether pollinator communities in natural systems are following similar trends, identifying factors which support pollinator visitation and services are important for ameliorating the effects of the current global pollinator crisis. We investigated how fire affects resource structure and how that variation influences floral pollinator communities by comparing burn versus control treatments in a southeastern USA old-field system. We hypothesized and found a positive relationship between fire and plant density of a native forb, *Verbesina alternifolia*, as well as a significant difference in floral visitation of *V. alternifolia* between burn and control treatments. *V. alternifolia* density was 44% greater and floral visitation was 54% greater in burned treatments relative to control sites. When the density of *V. alternifolia* was experimentally reduced in the burn sites to equivalent densities observed in control sites, floral visitation in burned sites declined to rates found in control sites. Our results indicate that plant density is a proximal mechanism by which an imposed fire regime can indirectly impact floral visitation, suggesting its usefulness as a tool for management of pollination services. Although concerns surround the negative impacts of management, indirect positive effects may provide an important direction to explore for managing future ecological and conservation issues. Studies examining the interaction among resource concentration, plant apparency, and how fire affects the evolutionary consequences of altered patterns of floral visitation are overdue.

## Introduction

The conservation and management of biodiversity is increasingly important to maintain vital ecosystem services [1], and anthropogenic changes to ecosystems continue to increase the risk of species extinctions across the planet [2,3]. Pollinators are one group of species that perform critical roles for the functioning of terrestrial ecosystems, provide important ecosystem services for natural plant communities, and influence global food security through agricultural products [4-9]. Specifically, more than 90% of angiosperms are dependent on animal pollination [10], and the economic value of pollination services for global agriculture is valued between $150 [8] and $200 billion [11]. Mounting evidence from agricultural ecosystems reveals significant honeybee (*Apis mellifera*) declines in many regions of the globe [12] and policies are addressing this issue in countries around the world. For example, domestic honeybee have been reduced by two thirds from 1947 to 2005 in the US [13], and declines of bumblebees (*Bombus* spp.) have been recorded across Europe [6,14,15], stimulating policy to reduce the use of specific neonicotinoid pesticides. Drivers of global pollinator declines are thought to include land-use changes (e.g. habitat loss, fragmentation, degradation, and pesticide usage), invasive species introductions and disease, climate change, as well as synergistic effects of multiples of these factors [9], yet it remains relatively unknown whether natural ecosystems are undergoing similar pollinator declines as in agricultural settings. Therefore, identifying factors that support or enhance floral visitation and pollination services is critical for ameliorating the effects of current global declines of pollinators. 

Maintaining sustainable pollination services requires an understanding of factors that influence pollinator visitation. Although relatively few studies have examined how pollinator communities are structured [16], such factors generally include abiotic and biotic environmental variation [17,18], suitability of and proximity to nesting habitats [19], and quantity and quality of forage resources (e.g. pollen and nectar) [20,21]. For example, the frequency of pollinator visitation in Ugandan coffee crops was most strongly correlated with forest distance, light intensity, and time of day [22], and interactions between temperature and pollen availability have been shown to influence pollinator visitation and subsequent plant fitness [23]. The genetic environment can also be important for determining pollinator visitation. For instance, Genung et al. (2010) found that as genetic diversity increased in patches of *Solidago altissima*, floral phenology shifted and floral abundance increased [24]. Furthermore, increases in floral resources had a strong positive effect on floral visitors. While the density of plants was similar from monoculture plots to mixture plots, the density of floral resources changed along this genetic gradient suggesting that plant density might be an important factor affecting floral visitation. Because variation in floral resources has important effects on broad patterns of floral visitation [25,26], identifying the factors that drive variation in those resources becomes important. 

In various fire-dependent communities, fire regimes have been shown to maintain or increase plant diversity [27–31], potentially affecting and increasing ecosystem services, typically by reducing vegetation, leaf litter cover, and woody encroachment that often promotes rare species establishment [32]. Components of fire regimes (e.g. fire frequency, severity, and intensity) can alter biotic and abiotic soil properties [33], as well as aboveground plant community structure and dynamics [29,34-36]. One study, which utilized two decades of low-severity prescribed burn treatments in a Midwestern oak forest (*Quercus* spp.), found that burned plots had greater pH and conductivity belowground, and greater canopy openness and herbaceous richness aboveground than unburned plots [37]. Moreover, old-field ecosystems contain plant communities that are largely structured by disturbances such as fire [38]. For example, McGinley and Tilman (1993) revealed that densities of old-field plant species were differentially affected by burn treatments and fire, but not other disturbances (i.e. tilling), which increased the density of *Euphorbia glyptosperma* [39]. Plant species in fire-dependent southeastern U.S. longleaf pine ecosystems have also shown increased flowering, fecundity, and seedling establishment in response to fire [31]. As a result, variation of post-fire plant communities might alter floral distributions and their resources (e.g. pollen and nectar), impacting pollinators and associated pollination ecosystem services.

The response of pollinator communities to floral resource density is complex, but can have important implications for plant fitness [40]. In general, there appears to be consensus regarding a positive relationship among floral resources and pollinator communities and activity [41]. For example, increasing floral density has consistently been correlated with greater pollinator diversity and abundance [16,26,42-44]. The resource concentration hypothesis may explain this phenomenon, as herbivore communities experience a positive response to plant densities [45]. Utilizing an old-field ecosystem in the southeastern U.S., we examined how fire as a common management practice might have indirect consequences for pollinator visitation by altering plant communities. We hypothesized that by changing plant resource levels, fire will affect pollinator visitation. This study was composed of distinct observational and experimental components to specifically test the prediction that the overall mechanism of fire regime will increase *V. alternifolia* density, and that increased *V. alternifolia* density is a proximal mechanism leading to an increase in pollinator visitation. Using this combined experimental approach, we show that altering plant density is the proximal mechanism by which an imposed fire regime can indirectly promote pollinator visitation.

## Methods

*Verbesina alternifolia* L. (Asteraceae) is a dominant species and abundant resource for pollinators in managed and unmanaged old-field ecosystems throughout the southeastern United States. This species is ideal to test the direct and indirect effects of fire on plant density patterns (direct) and pollinator communities (indirect) as its range stretches from Texas to Ontario, Canada [46] and can occur in areas where fire is used as a management tool to support native plant recruitment [47]. *V. alternifolia* is a perennial forb species with a composite inflorescence and is observed to interact with a range of flower-visiting arthropods, including Hymenoptera, Lepidoptera, Coleoptera, and Hemiptera species. Specifically, *V. alternifolia* has shown greater seed germination with heat exposure compared to co-occurring native species [48], indicating that prescribed fire may influence patterns of abundance in this species. Since *V. alternifolia* interacts with a variety of flower-visiting arthropods, changes in plant abundance could have consequences for the distribution of floral resources and indirectly affect pollinator communities. 

### Observational Study

In September of 2012, an observational survey was conducted in three paired old-field sites to understand how fire may directly influence plant density and, indirectly, influence floral communities. Sites with fire treatments were located in Cades Cove, Great Smoky Mountains National Park, Tennessee. The sites were approximately 0.2 to 0.4 hectares in size, geographically separated by 1.5 km and located where low-intensity, stand replacement controlled burn treatments were applied during the dormant season in 2009, 2010, or 2011 (henceforth, “burn”), with a paired non-burned area (henceforth, “control”). Two of the three burn sites have experienced previous fires in 2001 and 2002, respectively, one of which being a controlled burn and one a wildfire with similar stand replacement characteristics to the prescribed treatments. Inferring fire intensity by the response of vegetation, stand replacement fire regimes for old-field ecosystems are considered low-intensity since the majority of pre-existing vegetation responds quickly and impacts on the soil are minimal and transient. Long-term site disturbance histories are unknown (Great Smoky Mountains National Park, Rob Klein, *personal communication*). Information regarding and permission to access each study location was provided by Rob Klein (fire ecologist, Great Smoky Mountains National Park). No endangered or protected species were sampled in this study. Within each burn and control treatment, we established three randomly selected 1 m^2^ plots, spaced at least 3.5 m apart, to measure *V. alternifolia* density and pollinator visitation. At both burn and control sites, infrequent and reduced pollinator visitation to solitary *V. alternifolia* inflorescences was observed. As such, all sampled plots contained mature *V. alternifolia* plants (~1.5 m in height) having an adequate number of inflorescences to allow for pollinator visitation (i.e. > 2 flowers; MVN *personal observations*). Heterospecific plants were excavated and removed from plots to eliminate the potential effect of species interactions on pollinator visitation, and the density of *V. alternifolia* was recorded (number of stems m^-2^). Previous studies have quantified flower density as a metric of floral resource [26,40], and similarly we refer to the density of mature *V. alternifolia* as a floral resource for pollinators in this system. The random arrangement of plots within sites was used to control for the effect of adjacent plot density on pollinator visitation. We established observational plots one week before measuring pollinator visitations in order to standardize the timeframe and environmental conditions across the observational and experimental components of our study (see Experimental Manipulation). Five separate, random plots at the same sites were sampled for total plant richness (number of unique plant species 625 cm^-2^) in burn and control treatments to determine if there were differences in plant community composition between treatments and if this affected pollinator communities. 

We measured floral visitation by known pollinators at all plots within a four hour timespan (1100 to 1500 hours) in 10 randomly selected plots at each burn (n = 30) and control (n = 30) treatment sites. All arthropods visiting any *V. alternifolia* structure were identified to order and morphospecies in the field, including seven Hymenoptera, six Coleoptera, four Araneae, three Hemiptera, and two Lepidoptera species. The long disk florets of the composite inflorescence typically attract long-tongued bee pollinators and, as such, this study focuses on the dominant flower-visiting arthropods identified as the subset of pollinating arthropods: honeybees (Hymenoptera: Apidae), bumblebees (Hymenoptera: Apidae), and sweat bees (Hymenoptera: Halictidae). Floral visitation by pollinators was measured over three-minute intervals at each plot by a single observer; all observers received equal visitation and identification training prior to measurements. Four independent observers were randomized over plots after every observation period and present over a simultaneous time period recording floral visitation at separate plots in the study. Average weather conditions over the observation period were obtained from the National Park Service weather monitoring database and were recorded as 22° C, 74% relative humidity, 0.12 mm rainfall, 152 watts/m^2^ solar radiation, and wind at 1.7 m/s from the west. The abundance of pollinator visitations was measured as the total number of individuals that entered the plot and visited a *V. alternifolia* flower, regardless of how many independent inflorescences the pollinator visited within each plot [49].

### Experimental Manipulation

The observational results suggested that fire altered plant density indirectly impacting floral arthropod communities; therefore, we performed an experiment examining the hypothesis that *V. alternifolia* density was a mechanism by which prescribed burns may indirectly affect pollinator communities. To test this hypothesis, we standardized densities of three additional 1 m^-2^ plots randomly placed within all burn sites to control for the effect of surrounding vegetation on pollinator visitation. Observed *V. alternifolia* stem densities within the burn sites were reduced to equivalent densities that were found in control sites (~8 stems m^-2^) by excavating random individuals from each plot. Pollinator visitation was then measured with the same procedure as in the observation component of the study. To minimize the effects of removal disturbance on pollinator activity, the density manipulation (i.e. plant excavation) was performed one week prior to all pollinator visitation measurements. 

### Statistical Analyses

To test for the effects of fire on *V. alternifolia* density and floral pollinator visitation, we used a Restricted Estimated Maximum Likelihood (REML) mixed model. To test whether site influenced the effect of burn or control treatments on plant density or floral communities, we initially analyzed all response variables with site as a random effect; treatment and site × treatment interaction were considered as fixed effects. No significant interaction effect was found; as a result, the interaction term was removed from the model and the effect of fire on *V. alternifolia* density and floral communities was analyzed with treatment as a fixed factor and site as a random effect using JMP Pro 10 (SAS Institute, Cary, NC, USA). We used a meta-analysis approach to examine the effect of fire by comparing standardized z-scores between burn and control treatments. Although some studies have indicated that fire can affect floral richness with implications for pollinator communities [25,50], we did not find evidence of fire affecting subsequent total plant richness in the plots, nor were pollinators influenced by total plant diversity (Figure S1a-c). 

## Results

### Observational Study

**Figure 1:**
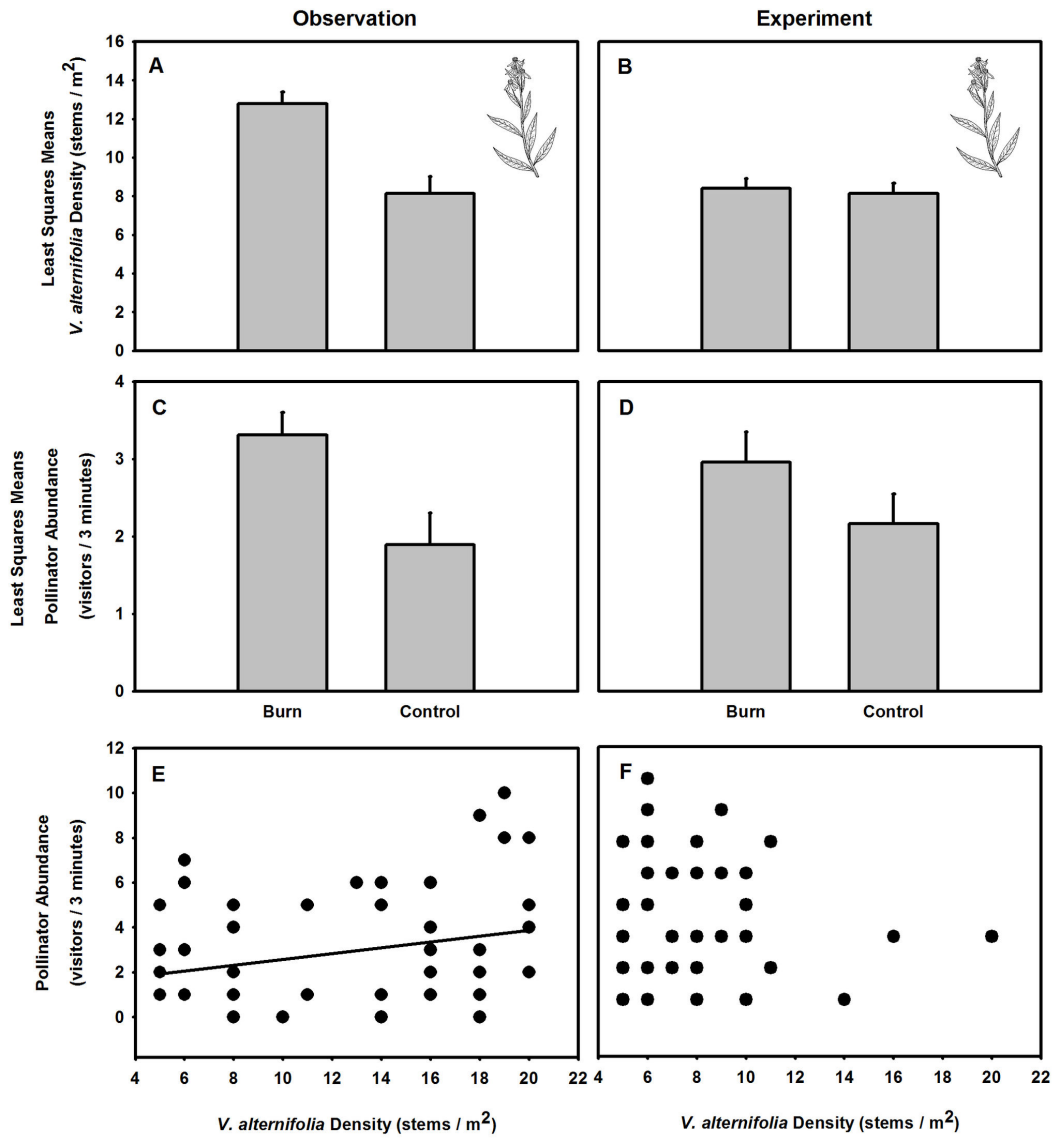
Observational and experimental impacts of fire on plant density and floral visitation. Comparisons from the observation (left panels, A, C, E) and experiment (right panels, B, D, F) show plant density is a mechanism by which fire impacts the abundance of pollinators. Bars represent least square means ± 1 SE.

Across all sites, fire positively affected *V. alternifolia* density and pollinator abundance. *V. alternifolia* density was 44% greater in burned treatments relative to control plots (Figure **1a**; Table **1**). Overall, pollinator visitation to *V. alternifolia* occurred 54% more frequently in burned treatments relative to control sites (Figure 1c; Table 1). Consistent with the hypothesis that fire indirectly impacts floral pollinator visitation by altering the density of *V. alternifolia*, on average there was a positive relationship between plant density and floral pollinator visitation (Figure 1e; r^2^ = 0.154, p = 0.018). 

**Table 1:** Restricted estimated maximum likelihood results (REML) for the effect of fire on *Verbesina alternifolia* density and pollinator abundance by study component.

Response Variable	Burn Treatment	Study Component
Density	F_(1)_=528.81; p<0.0001	Observational
Pollinator Abundance	F_(1)_=6.23; p<0.0156	Observational
Density	F_(1)_=0.86; p=0.357	Experimental
Pollinator Abundance	F_(1)_=0.495; p=0.484	Experimental

There were no significant site x treatment interaction effects so they were removed from the model.

### Experimental Manipulation

**Figure 2:**
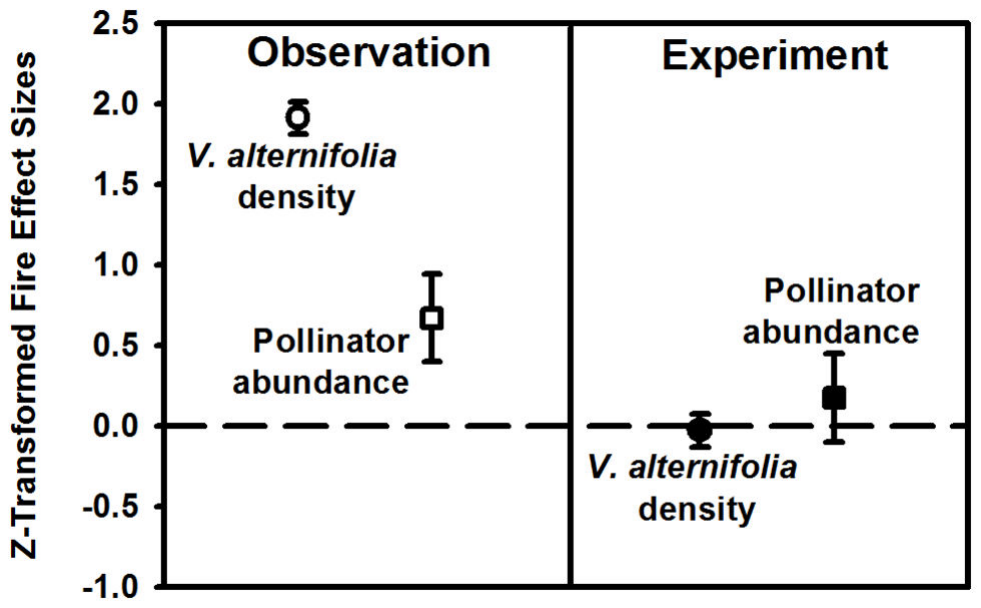
Standardized effects of fire on plant density and pollinator abundance. (Observation) Plant density and pollinators are positively affected by fire, but the effect diminishes from plants to pollinators. (Experiment) Manipulated and equivalent plant densities between burn and control treatments shows no effect of fire on pollinator abundance. Boxes represent means of standardized z-scores, and values greater than zero indicate a positive effect of fire. Error bars show ± 1 SE and, when non-overlapping with zero, indicate significant differences.

Observational results suggest that *V. alternifolia* density is a mechanism by which fire indirectly impacts pollinators. To test this hypothesis, densities of *V. alternifolia* in burn sites were successfully reduced to densities observed in control sites (Figure 1b; Table 1). Across all sites, when the density of *V. alternifolia* was controlled, no significant difference in floral pollinator visitation was observed between burn and control treatments (Figure 1d; Table 1), and no relationship existed between plant density and floral pollinator visitation. *V. alternifolia* responded 96% more strongly to fire than floral pollinator visitors directly (Figure **2**) confirming that fire can indirectly impact patterns of floral pollinator visitation and potentially pollination services by altering *V. alternifolia* density. 

### Pollinator community responses

**Figure 3:**
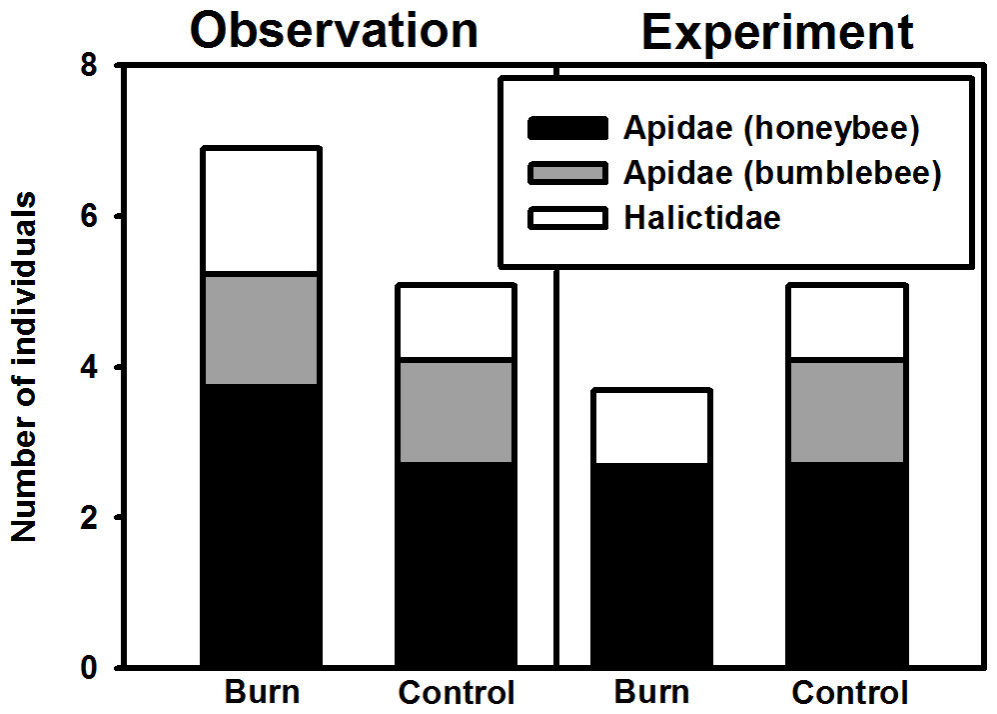
Fire treatment and study component influence pollinator-specific visitation patterns. Means of individuals from the three most common pollinators are depicted for both the observational study and experimental manipulation. Note floral pollinator visitation to control plots is identically represented in both studies, and bars depict individual averages over the visitation surveys.

Floral visitation by individual pollinators was differentially affected by treatments across study components (Figure **3**), indicating species-specific responses to fire and subsequent alterations of *V. alternifolia* density. During the observational study, honeybees showed a marginally significant 32% increase in abundance in burn versus control sites (p = 0.078). Interestingly, with equivalent plant densities during the experimental manipulation, honeybee visitation did not differ between burn and control treatments (p = 0.989), and bumblebees were entirely absent from experimental plots in burn sites. Additionally, sweat bee visitation was not influenced by fire or plant density treatments (observation: p = 0.220, experiment: 0.424). 

## Discussion

 Identifying factors that influence floral visitation and pollinator dynamics may aid efforts to mitigate global pollinator declines and establish sustainable ecosystem services provided by pollinators in a changing world [9]. We hypothesized that an imposed fire regime, as a management tool for plant communities, would have indirect consequences for pollinator communities through altered floral resource patterns. Our study shows that fire, with landscape level disturbance effects, may be a valuable tool for promoting pollination services and we provide evidence that altered plant density is a proximal mechanism by which a fire regime indirectly impacts pollinator visitation. Moreover, species-specific responses by the most prevalent pollinators suggest different groups may be more or less sensitive to the effects of fire on altered floral resource densities. Indirect but positive consequences of management regimes, a relatively unexplored topic, may be an effective and important approach for acting on future ecological and conservation issues.

Fire is a landscape level process that structures plant [51] and insect [52] communities from local to regional scales. Fire has also been shown to influence patterns of herbivory [53,54], trophic interactions [55,56], and overall arthropod community structure [52,53,55]. While plant richness and community composition was largely unaffected, our study found that burning significantly increased the density of *V. alternifolia*. These results are consistent with many studies that have shown positive species-specific responses to fire. For example, aspen trees (*Populus tremuloides*) responded positively to fire by increasing densities of ramet resprouts [53]. Specific to old-field ecosystems, previous work has shown plant species are affected by fire differently, and greater post-fire densities may be due to differences in establishment ability [39]. One potential explanation for the positive response of *V. alternifolia* to fire is a significant increase in seed germination when exposed to heat treatments compared to co-occurring native species, likely an adaptation to naturally occurring fire regimes [48]. In combination with increased availability of mineral nutrients and light immediately following fire [57,58], fire might influence the observed patterns of *V. alternifolia* density by interacting with this early life history stage, similar to other plant community dynamics following disturbance in old-field ecosystems. In addition to changes in plant density, the effects of fire extended to the pollinator community by increasing floral visitation suggesting that fire may promote pollination services in some systems. Previous studies have documented a similarly positive indirect effect of fire on pollinators [59,60]. For example, bee abundance has been shown to track plant communities following fire. This has largely been explained as a post-fire relationship between pollinators and nectar reward structure (e.g. floral reward energy) [16,25]; however, these studies lack experimental verification of mechanisms. Here we show that shifts in plant density are an important local mechanism that can change with fire and indirectly impact floral visitation.

Fire may serve as an important tool for mitigating pollinator declines by influencing plant communities as predicted under the resource concentration hypothesis. Plant apparency is a common hypothesis invoked to understand patterns of herbivory and floral visitation by arthropods [61,62]. Resource concentration, as a distinct component of plant apparency by operating at a local scale, predicts that larger or denser stands of plants (i.e. concentrated resources) will support greater abundance of herbivores per plant [45,63]. For example, manipulated strawberry patch size and density were shown to increase *Lygus lineolaris* nymph abundance per inflorescence [64]. The majority of cases investigating the resource concentration hypothesis have focused on specialist herbivore-plant interactions [64]. We extend this framework to pollinator-plant interactions by showing that higher densities within floral patches increased pollinator visitation. While previous studies have indicated that local plant density can influence pollinator dynamics [41], we provide experimental verification of density as a mechanism impacting pollinator abundance. Our study shows that fire, operating indirectly on pollination services through plant apparency and resource concentration, may be effective in ameliorating declines of pollinators and sustaining their ecosystem services in old field ecosystems.

Because fire alters both plant apparency and resource concentration, these factors likely interact to influence pollinator visitation. Though we did not measure it directly in this study, the frequency of high density patches was likely greater in burn sites than in control sites, making floral resources both more apparent within sites to floral visitors and more concentrated at a local scale. The observed species-specific pollinator visitation patterns may be a consequence of this interaction. In our study, bumblebee visitation responded more strongly to altered plant apparency (i.e. no visitation to experimental burn sites containing fewer high density patches of *V. alternifolia*), whereas honeybee visitation appeared to follow resource concentration (i.e. greater in burn versus control sites during the observational study, but no difference during experimental manipulation). Alternatively, species-specific visitation patterns may be attributed to differences in pollinator behavior and life history. For instance, the prevalence of non-native honeybees is predicted to have deleterious effects on native bee pollinators due to competition [65], while bumblebees are common nectar robbers [66] and sweat bees are often generalist foragers [67]. The prevalence of honeybees in this study, as well as the behavioral traits of the other dominant pollinators, may have played a role in how individual pollinator groups responded to variation in floral resource distributions. To understand how fire regime, plant apparency, and resource concentration interact to influence specific pollinator visitation, future work should manipulate patch number as well as within-patch *V. alternifolia* density across burned and control management areas while focusing on behavioral differences between dominant pollinator species.

### Conclusions and implications

Consistent with the resource concentration hypothesis, our study provides evidence that an imposed fire regime indirectly and positively impacts pollinators by altering plant density. These data suggest that future studies would benefit from examining the interactions among fire, plant apparency, and resource concentration, in addition to the extended consequences of increased floral visitation on plant fitness to understand how fire may influence the evolutionary dynamics of species through plant-pollinator interactions. Lastly, although negative indirect effects of management regimes may occur, indirect positive effects may provide an important management direction for future ecological and conservation issues - especially considering previous studies have observed positive effects of components of fire regimes on pollinators and warrants much more research attention. 

## Supporting Information

Figure S1 **Fire did not affect, and pollinators were not affected by, plant community richness or composition.** (A) Plant richness is not different between burn and control treatments (F = 3.55, p = 0.07, bars represent means ± 1 SE). (B) Plant richness is not correlated with pollinator visitation rates (r^2^ = 0.060, p = 0.231). (C) Non-metric multidimensional scaling (NMDS) shows no difference in plant community composition between burn and control treatments (ANOSIM, Global R = 0.011, p = 0.336, centroids depict means ± 1 SE).
(TIF)
