# Supplementary figures and images for: Fire Promotes Pollinator Visitation: Implications for Ameliorating Declines of Pollination Services
Source: PLoS One. 2013 Nov 12;8(11):e79853. doi: 10.1371/journal.pone.0079853 (PMC3827174; doi:10.1371/journal.pone.0079853)

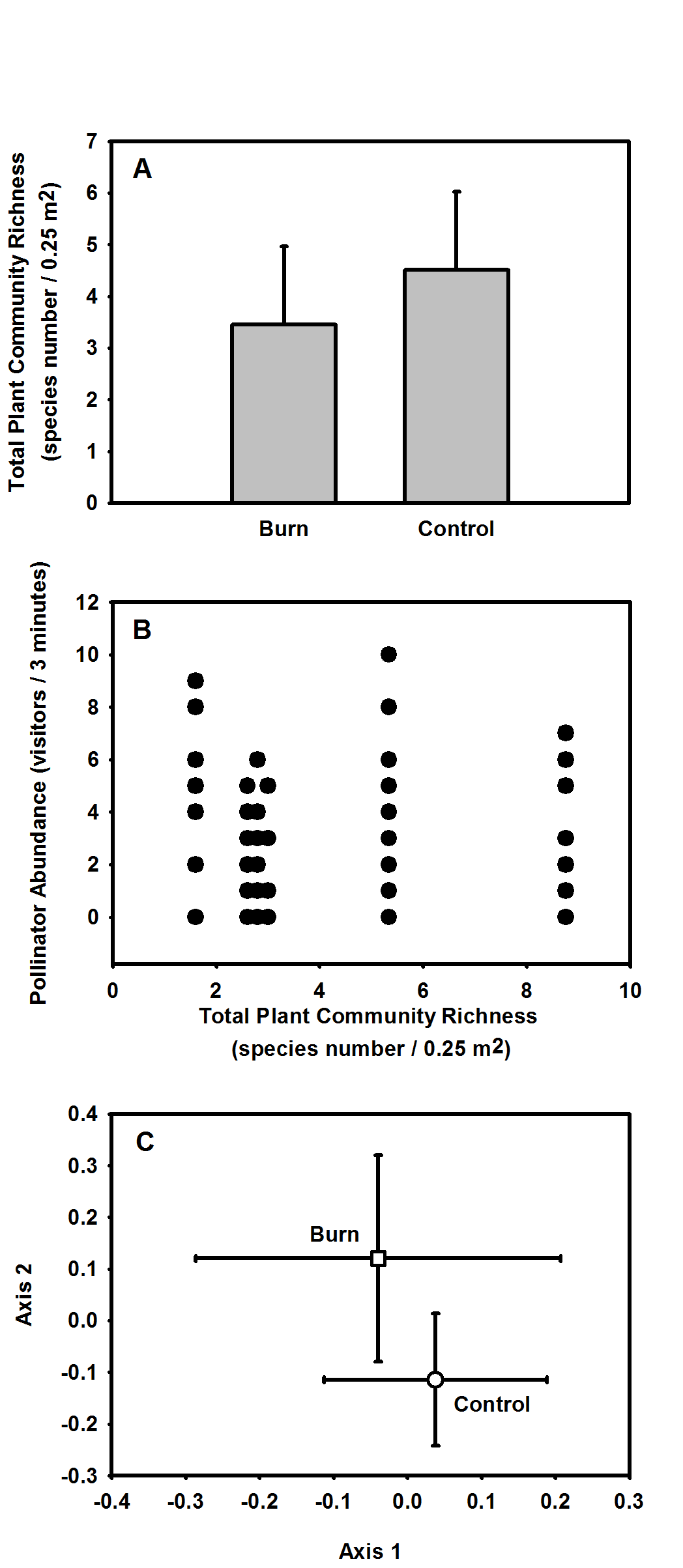

Supplement: Figure S1 — Fire did not affect, and pollinators were not affected by, plant community richness or composition. (A) Plant richness is not different between burn and control treatments (F = 3.55, p = 0.07, bars represent means ± 1 SE). (B) Plant richness is not correlated with pollinator visitation rates (r2 = 0.060, p = 0.231). (C) Non-metric multidimensional scaling (NMDS) shows no difference in plant community composition between burn and control treatments (ANOSIM, Global R = 0.011, p = 0.336, centroids depict means ± 1 SE). (TIF) [file pone.0079853.s001.tif]
